# A 36-Year Retained T-tube Fragment Presenting with Cholangitis

**DOI:** 10.4021/gr2009.04.1285

**Published:** 2009-03-20

**Authors:** Tarun Sharma, Katie F. Farah

**Affiliations:** a Division of Gastroenterology, Allegheny General Hospital, 320 East North Avenue, Pittsburgh, PA 15121, USA

**Keywords:** Retained T-tube, Cholangitis, cholecystectomy

## Abstract

The use of a T-tube to drain the biliary tree after choledochotomy has been a common surgical practice. Inadvertent fracture of the T-tube limb during removal is a rare occurrence which can lead to several complications. We report a case of cholangitis caused by a T-tube fragment retained in the common bile duct 36 years after cholecystectomy.

## Case Report

**Figure 1:**
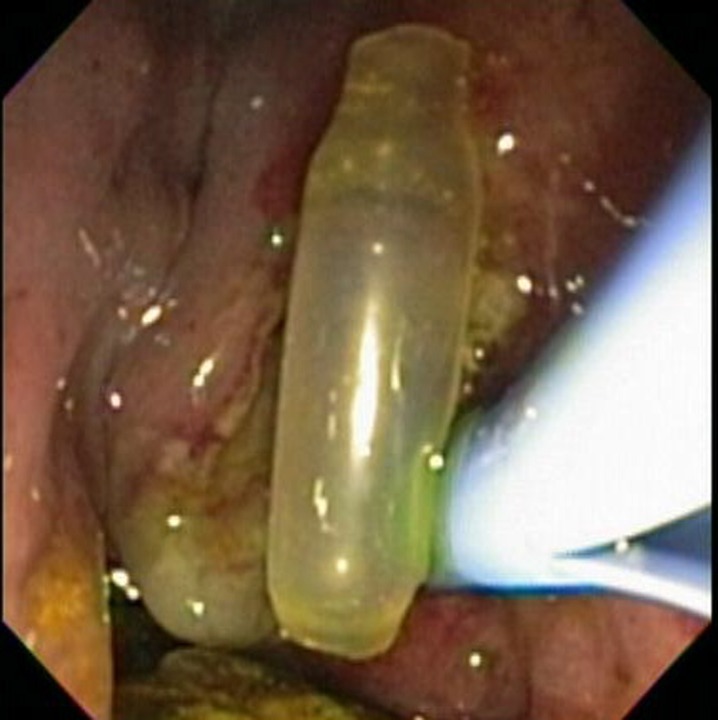
T-tube fragment.

A 79-year-old woman with a history of cholecystectomy and T-tube placement 36 years ago presented to our institution for evaluation of fever, abdominal pain, and elevated liver function tests. Physical examination was significant for jaundice and right upper quadrant tenderness. Laboratory investigation revealed AST 54 U/L, ALT 140 U/L, AP 221 U/L, TBil 4 mg/dL and WBC 15000/mm^3^. MRCP revealed severe intrahepatic and extrahepatic biliary dilatation and choledocholithiasis. ERCP showed a bulging papilla, common bile duct 1.5 cm and choledocholithiasis. A large biliary sphincterotomy and sphincteroplasty were performed. A 15 mm balloon was used for stone extraction and a significant amount of exudate was visualized. A clear plastic tubular object was visualized in the distal bile duct which upon balloon extraction proved to be a T-tube remnant (Fig. 1).

## Discussion

Insertion of a T-tube post-choledochotomy has been the traditional method used to drain the biliary tree. It is still considered a useful tool in biliary surgery despite the decreasing number of bile duct explorations and the trend towards performing primary closure of a choledochotomy. Historically, decompression of the biliary tree was preferred to allow spasm and edema of the sphincter to settle, to obtain postoperative cholangiography to detect retained stones and to develop a tract along which percutaneous removal of a retained stone could be achieved [1]. Complicated cases with obstructive jaundice, septic cholangitis, pancreatitis, or difficult CBD exploration may all warrant T-tube insertion [1]. However, the use of these tubes has been associated with several complications. Morbidity occurring with the T-tube in situ includes fluid and electrolyte disturbances, sepsis, premature dislodgement, and bile leakage [2]. Bile leakage after removal occurs more frequently (1-19%), which can lead to bile ascites, biloma, or bile peritonitis [2]. Other complications include bile duct trauma, prolonged fistula, and stricture formation.

Retention of a T-tube fragment upon removal is a rare complication. A review of the literature shows only 9 reported cases of a T-tube fragment retained in the common bile duct. In 5 of these cases, the patients presented with cholangitis and had retained a fragment for 2 to 9 years [3-7]. The fragment was removed by ERCP with sphincterotomy in 4 cases and surgically in one case. In 4 cases the tube was known to have fractured during its removal and therefore immediate removal was possible [8-11]. Of these, 2 fragments were removed by ERCP with sphincterotomy, one using a percutaneous approach with a balloon catheter, and one with intra-operative duodenoscopy.

We report a rare case of choledocholithiasis and subsequent cholangitis caused by an accidentally retained T-tube fragment occurring 36 years after cholecystectomy. This is the longest reported duration of a retained T-tube. Additionally, our case illustrates that such patients may be asymptomatic for years until complications develop. Cholestasis secondary to foreign body obstruction and local inflammatory response from the plastic are presumed etiologic factors in the pathogenesis. An ERCP with sphincterotomy is an appropriate initial step in management. Although this is an uncommon occurrence, it is important to ensure that the T-tube be removed intact to avoid potential serious complications.
